# Changes in protein expression during honey bee larval development

**DOI:** 10.1186/gb-2008-9-10-r156

**Published:** 2008-10-29

**Authors:** Queenie WT Chan, Leonard J Foster

**Affiliations:** 1 Centre for High-Throughput Biology, Department of Biochemistry and Molecular Biology, University of British Columbia, Vancouver, BC, V6T 1Z4, Canada

## Abstract

**Background:**

The honey bee (*Apis mellifera*), besides its role in pollination and honey production, serves as a model for studying the biochemistry of development, metabolism, and immunity in a social organism. Here we use mass spectrometry-based quantitative proteomics to quantify nearly 800 proteins during the 5- to 6-day larval developmental stage, tracking their expression profiles.

**Results:**

We report that honey bee larval growth is marked by an age-correlated increase of protein transporters and receptors, as well as protein nutrient stores, while opposite trends in protein translation activity and turnover were observed. Levels of the immunity factors prophenoloxidase and apismin are positively correlated with development, while others surprisingly were not significantly age-regulated, suggesting a molecular explanation for why bees are susceptible to major age-associated bee bacterial infections such as American Foulbrood or fungal diseases such as chalkbrood. Previously unreported findings include the reduction of antioxidant and G proteins in aging larvae.

**Conclusion:**

These data have allowed us to integrate disparate findings in previous studies to build a model of metabolism and maturity of the immune system during larval development. This publicly accessible resource for protein expression trends will help generate new hypotheses in the increasingly important field of honey bee research.

## Background

Honey bees (*Apis mellifera*) have been a subject of scientific research for more than 2,300 years [1], yet it is only in the past two decades that bee research has expanded beyond behavioral or social traits to a molecular level. With the publication of the honey bee genome in 2006 [2], the basic information to enable proteome-level analyses of this organism is now available. Since then, various groups have published proteomic analyses of whole bees or individual organs/tissues [3-6] but these studies have focused on adult animals. Larval development in honey bees is largely unexplored, despite its significance in caste determination [7] and in the pathogenesis of certain economically significant honey bee diseases, such as American and European Foulbrood.

The larval development of the honey bee, which follows a 3-day period as an egg, is 5-6 days in duration and precedes the pupal (metamorphosis) and adult stages. Apart from an astounding increase in size, larval growth is relatively unremarkable at the macroscopic level [8]. However, female bees differentiate into workers or queens (caste differentiation) in response to diet very early in larval development and the acquisition of immunity to certain diseases during this 5- to 6-day period suggests complex molecular biological changes are taking place.

Insect development has been studied mainly using the fruit fly as the model system. *Drosophila *embryogenesis has historically attracted far more attention than any other growth stage, due to its value for studying the mechanism of spatial regulation of transcription and translation. With the exception of the economically important silkworm *Bombyx mori*, research on larval development has been slow. For honey bees, the lack of published works is evident: the article entitled 'Morphology of the Honeybee Larva' published by Nelson in 1924 [8] still remains today as one of the most cited resources on this subject. Here we have used mass spectrometry-based proteomics to profile the changing abundance of individual proteins over the first 5 days of the worker larval stage and used these data, with the help of sequence-based function prediction, to build a framework for the developmental processes going on in the maturing larva.

## Results

**Figure 1:**
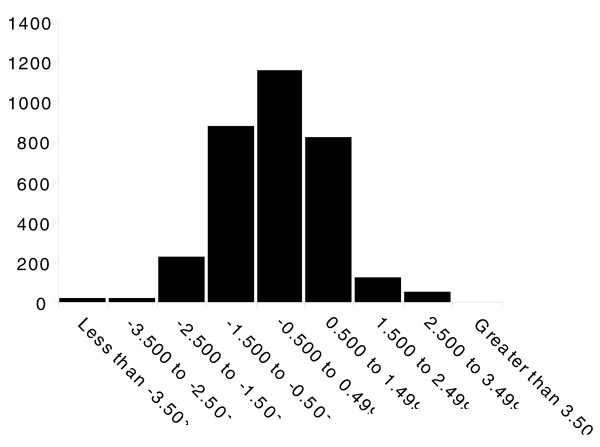
The peptide ratios within an experiment are roughly normally distributed and show no labeling bias. Using replicate number 1 of day 1 versus day 3 solid tissue quantification data as an example, the peptide ratios are displayed as a histogram, sorted into natural-log unit bins (bin size = 1).

In order to obtain suitably aged larval samples for proteomic profiling of the first 5 days of development, for each experiment we isolated an open-mated, laying queen on an empty frame of brood comb for a short period of time to allow her to lay several hundred eggs (see Materials and methods). The frame and queen were then separated by a queen excluder and workers were allowed to tend the brood. Starting on the day the eggs hatched (day 1, roughly corresponding to first instar) larvae were collected every day for 5 days. Hemolymph was separated from the remaining tissues (termed 'solid tissues' henceforth) prior to protein extraction (see Materials and methods) and equal amounts of protein from each age were resolved on a reducing SDS polyacrylamide gel (Figure 1). The protein composition of solid tissues was grossly consistent across all ages, but varied drastically in the hemolymph. Hemolymph from 1- to 3-day old larvae show a staining pattern distinct from that of 4- to 5-day old larvae. These differences may be partially attributed to slight variations in collection methods for young and old larvae but it is more likely that these represent real biological changes occurring as the late larvae prepare for pupation. Most notably, a 70 kDa hexamerin band emerges from day 3 and beyond and accounts for the majority of the protein in the hemolymph, an observation that has been made numerous times by other researchers [9-11]. A second observation that argues against these dramatic changes around day 3 being simply an artifact of sample collection is the absence of the major protein bands in the hemolymph gel in the solid tissue gel, and vice versa.

As a means for identifying and quantifying the expression profiles of proteins in developing larvae, we used a quantitative proteomics approach employing stable isotope labeling and liquid chromatography-tandem mass spectrometry. The labeling method we used employs deuterated and hydrogenated forms of formaldehyde to reductively dimethylate primary amines in peptides, but since there are only two labeling conditions possible in this schema, we compared the expression of protein from days 1, 2, 4 and 5 larvae versus that from day 3 in order to generate an expression profile spanning the whole development period. Three biological replicates of each tissue type were analyzed, which resulted in the detection of 12,421 non-redundant peptides (supplementary Table 10 in Additional data file 1). After applying the cutoff criteria (see Materials and methods), 1,333 proteins were identified (supplementary Table 1 in Additional data file 1) with an estimated false discovery rate of 0.97% (see Materials and methods), thus providing experimental evidence for 12.7% of the 10,517 genes in the predicted honey bee gene set. In general, the peptide ratios showed no labeling bias and were approximately normally distributed (Figure 1). Among these, 790 were quantified in 2 or more days by averaging the intensity ratio from at least 2 of the 3 replicates (if more than 5 peptides were quantified, the top 5 most intense peptides were selected): 378 (48%) of them matched this criterion in both the tissue and hemolymph, 309 (39%) were specific to solid tissue and 103 (13%) were specific to hemolymph. An example of using peptide ratios to derive relative protein expression profiles is shown in Table 1 for the odorant binding protein 14 [GenBank:94158822].

**Table 1:** An example of using peptide ratios used to derive protein relative expression results (odorant binding protein 14 [GenBank:94158822])

			Peptide						
					
Sample	Larval ages compared	Replicate (1, 2, or 3)	1	2	3	4	5	Ln (peptide average)	Ln (protein average)
H	1, 3	1	-1.26	-1.41	-1.66	-1.82	-2.25	-1.68	-3.01
H		2	-3.29	-3.39	-3.55	-3.68	-3.74	-3.53	
H		3	-3.76	-3.82	-3.82	-3.82	-3.82	-3.81	
									
H	2, 3	1	-0.68	-0.93	-1.28	-1.32	-1.77	-1.20	-1.35
H		2	-0.85	-0.97	-1.06	-1.13	-1.62	-1.12	
H		3	-1.57	-1.59	-1.69	-2.11	NA	-1.74	
									
H	4, 3	1	3.91	3.91	3.91	3.91	NA	3.91	2.22
H		2	-0.10	0.53	0.91	1.94	3.10	1.28	
H		3	0.67	1.56	1.60	1.69	1.84	1.47	
									
H	5, 3	1	3.91	3.91	3.91	3.91	3.91	3.91	3.09
H		2	2.16	2.32	2.50	3.04	3.04	2.61	
H		3	2.05	2.55	2.57	2.78	3.79	2.75	
									
T	1, 3	1	-1.29	-1.37	-1.57	-1.80	-1.80	-1.57	-0.53
T		2	-1.68	-2.16	-2.16	NA	NA	-2.00	
T		3	1.47	1.83	2.59	NA	NA	1.96	
									
T	2, 3	1	-0.55	-0.58	-0.78	-0.91	-0.93	-0.75	-0.72
T		2	-0.34	-0.42	-0.55	-1.04	-1.42	-0.75	
T		3	-0.46	-0.66	-0.86	NA	NA	-0.66	
									
T	4, 3	1	-0.39	-0.40	-0.69	-0.70	-0.76	-0.59	-0.37
T		2	-0.06	0.24	0.38	0.70	0.81	0.41	
T		3	-0.65	-0.95	-1.02	-1.07	NA	-0.92	
									
T	5, 3	1	-0.02	-0.05	-0.27	-0.31	-0.40	-0.21	0.76
T		2	0.37	0.67	0.90	1.47	NA	0.85	
T		3	0.80	1.53	1.67	2.04	2.08	1.62	

Both the honey bee larval hemolymph (H) and tissue (T) samples were collected daily for 5 days post-hatching, and peptides from days 1, 2, 4, and 5 were isotopically labeled and mixed at 1:1 (by protein amount) with day 3 peptides, which were labeled differentially from the other days. Relative peptide intensities were recorded (limited at 50-fold or 3.91 in natural log (Ln)) and proteins with a minimum of 2 quantified peptides were natural log-transformed and averaged; for proteins with greater than 5 peptides, the top 5 most intense ones were selected for averaging. In samples where there were less than 5 peptides, their absence is indicated by NA (not available).

**Figure 2:**
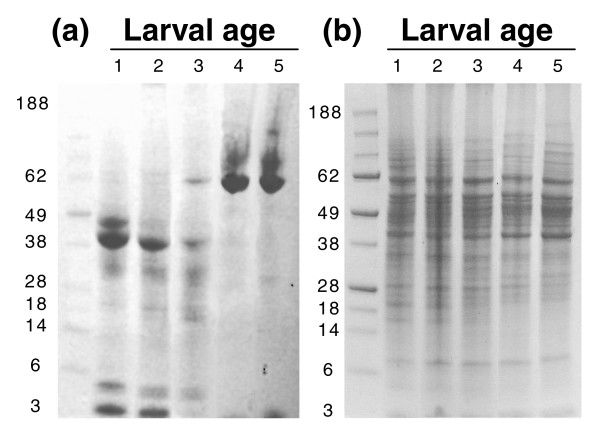
PAGE of honey bee larvae **(a) **hemolymph and **(b) **solid tissue. Age is shown in days post-hatching. Molecular weight markers are shown on the left.

**Figure 3:**
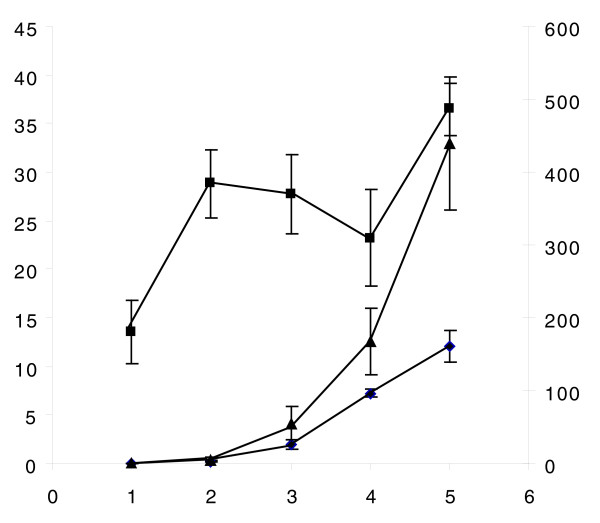
Developmental changes of larval hemolymph. The left axis denotes the volume of hemolymph per larva (diamonds; μl) or hemolymph protein concentration (squares; μg/μl), while the right axis describes the mass of total protein per larva (triangles; μg). Measurements were made by pooling 5-120 larva (n = 3 separate pools) depending on age (x-axis, in days) and size. (Error bars represent 2 standard deviations.)

A major strength of this method is the ability to track the changing abundances of hundreds of proteins during development. Those whose levels can be traced for at least 4 out of 5 days in either the tissue or hemolymph were considered to have an informative profile, a total of 522 proteins. Approximately equal numbers of tissue proteins showed an expression trend either positively or negatively correlated with age, but the latter was more common for hemolymph proteins, as might be expected from the high dynamic range of hemolymph as shown in Figure 2. It is crucial to note that the decreasing trend likely does not reflect an absolute reduction in expression levels of most proteins, but is rather a phenomenon of analyzing equal amounts of protein between two samples with a very large difference in absolute protein amounts caused primarily by drastic increases in secreted hexamerins. Consequently, lower abundance proteins become harder to detect in this background. Although the protein concentration in hemolymph changes only slightly beyond 1 day after hatching, the total volume, and thus absolute protein content, increases exponentially with age (Figure 3).

There is no direct functional information available for more than 99% of honey bee proteins, so to derive some functional insight from the data acquired here we used BLAST2GO [12] to systematically predict function based on sequence similarity (supplementary Table 2 in Additional data file 1). After grouping specific molecular function ontologies into broader categories until they converged under one term (supplementary Table 3 in Additional data file 1), the third-level terms were analyzed in detail. To find whether a given function term was developmentally regulated, an average expression profile was generated using data from proteins belonging under each term and tested for significance at the *p *< 0.05 level (see Materials and methods). The slope between day 1 and day 5 was calculated to approximate the directionality and strength of temporal correlation. In the 34 terms considered, 11 of them had activity profiles that satisfied the significance criteria in at least one of either the solid tissue or hemolymph expression profiles (Table 2; details in supplementary Table 4 in Additional data file 1). Gene Ontology (GO) terms 'substrate-specific transporter activity' [GO:0022892] and 'transmembrane transporter activity' [GO:0022857], both of which were tissue-specific activities, were very mildly positively correlated with larval age. The majority were negatively correlated with age, with the most statistically significant being 'structural constituent of ribosome' [GO:0003735] and 'nucleic acid binding' [GO:0003676]. Others showing a similar trend include 'enzyme inhibitor activity' [GO:0004857], 'helicase activity' [GO:0004386], and 'nucleotide binding' [GO:0000166]. Terms that did not show regulation in either the tissue or hemolymph tended to be ones with non-specific participation in different pathways, such as 'transferase activity' [GO:0016740], 'kinase regulator activity' [GO:0019207] and 'cofactor binding' [GO:00048037].

**Table 2:** Expression trends of proteins categorized under Gene Ontology terms

Organ	GO ID number	GO term	Proteins considered	*t*-Test of slope between day 1 and day 5	Slope
H	GO:0004857	Enzyme inhibitor activity	6	1.9E-02	-0.19
T	GO:0004857	Enzyme inhibitor activity	6	0.007	-0.41
T	GO:0004386	Helicase activity	4	0.016	-0.37
T	GO:0016787	Hydrolase activity	80	0.002	-0.10
H	GO:0003676	Nucleic acid binding	18	7.2E-05	-0.24
T	GO:0003676	Nucleic acid binding	38	7.8E-09	-0.33
H	GO:0000166	Nucleotide binding	36	4.8E-03	-0.09
T	GO:0000166	Nucleotide binding	72	0.022	-0.08
H	GO:0016491	Oxidoreductase activity	19	1.8E-02	0.18
H	GO:0004871	Signal transducer activity	3	4.7E-02	-0.22
H	GO:0003735	Structural constituent of ribosome	18	1.5E-08	-0.41
T	GO:0003735	Structural constituent of ribosome	35	8.4E-16	-0.34
T	GO:0022892	Substrate-specific transporter activity	34	0.035	0.11
T	GO:0008135	Translation factor activity, nucleic acid binding	10	0.037	-0.24
H	GO:0022857	Transmembrane transporter activity	4	3.9E-02	-0.11
T	GO:0022857	Transmembrane transporter activity	27	0.010	0.12

Proteins were categorized under third-level molecular function terms and were evaluated as a group to assess whether their expression trends were age-regulated by performing paired *t*-tests comparing values from day 1 and day 5 of larval development, reporting the average slope between these two days if *p *< 0.05 in either the solid tissue (T) or hemolymph (H). The total number of proteins belonging under a particular GO term considered in the calculation is listed under 'Proteins considered'.

With the current lack of a thoroughly curated protein function database for the honey bee, we manually assigned functional categories by employing a variety of available bioinformatic tools (see Materials and methods, and supplementary Table 5 in Additional data file 1). This is necessary because certain major classes of honey bee proteins, such as hexamerins and odorant binding proteins, do not have high enough homology to proteins in other better annotated organisms and would thus be ignored. Furthermore, most proteins were assigned to multiple terms, or two very similar proteins were assigned to different but similar terms ('nucleic acid binding' [GO:0003676], and 'translation factor activity, nucleic acid binding' [GO:0008135]), which greatly complicates downstream hierarchical clustering and enrichment analysis. Groups that showed a significant temporal regulation (criteria nearly identical to the analysis of level 3 molecular function GO terms) are shown in Table 3 (details in supplementary Table 6 in Additional data file 1). A common protein expression pattern within a group was frequently observed. Ribosomal protein levels in the tissue were consistently lowest at day 2 and day 5, but overall decreased in relative concentration with age (*p *< 1e-16). Proteasome subunits and protein-folding chaperones exhibited the same overall trend (*p *< 1e-9 and *p *< 0.005, respectively). Energy storage proteins, including apolipoproteins and hexamerins, increased with age throughout the body but the trend was more dramatic in the hemolymph (*p *< 0.005). There were no signs of temporal regulation of enzymes for fatty acid synthesis, beta oxidation, and carbohydrate metabolism. However, several groups of energy producing proteins showed varying degrees of positive correlation with time: tricarboxylic acid cycle proteins (*p *< 0.05), ATP synthase subunits (*p *< 0.0005), and electron transport chain enzymes (*p *< 0.00005). Surprisingly, we observed a decreased expression of antioxidant proteins, members of the Ras GTPase superfamily, and ubiquitylation enzymes in the solid tissues as development progressed (*p *< 0.05, *p *< 0.01, and *p *< 0.05, respectively). Many typically intracellular proteins, such as ribosomal proteins and proteasome subunits, were found in hemolymph as we have described previously [3] and as others have reported in other insects [13,14].

**Table 3:** Expression trends of manually annotated and categorized proteins

Organ	Class	Proteins considered	*t*-Test of slope between day 1 and day 5	Slope
T	Adaptor	2	0.002	-0.43
T	Aldo-keto reductase superfamily	3	0.041	-0.22
T	Antioxidant	15	0.017	-0.20
T	ATP synthase	10	1.4E-04	0.21
H	Carbohydrate metabolism	15	0.003	0.20
T	Cuticle	7	0.036	0.19
T	Electron transport chain	14	1.1E-05	0.22
H	Energy storage	4	0.004	1.20
T	Energy storage	5	0.028	0.56
T	Kinases or phosphatases	2	0.044	0.24
H	Pentose phosphate pathway	4	0.001	0.06
H	Peptidase	15	0.045	0.14
H	Proteasome	9	1.4E-04	-0.23
T	Proteasome	18	8.6E-10	-0.32
T	Protein folding	34	0.001	-0.17
T	Ras superfamily	10	0.009	-0.27
T	Ribonucleoprotein	4	0.024	-0.43
H	Ribosome	20	2.1E-09	-0.40
T	Ribosome	38	4.7E-17	-0.34
T	Tricarboxylic acid cycle	21	0.033	0.10
T	Translation	14	0.015	-0.25
T	Ubiquitination	3	0.021	-0.35
H	Uncategorized	21	0.029	0.18

Proteins were categorized manually by function and evaluated as a group to assess whether their expression trends were age-regulated by performing paired *t*-tests to compare values from day 1 and day 5. Significant (*p *< 0.05) groups in either the solid tissue (T) or hemolymph (H) are shown.

**Figure 4:**
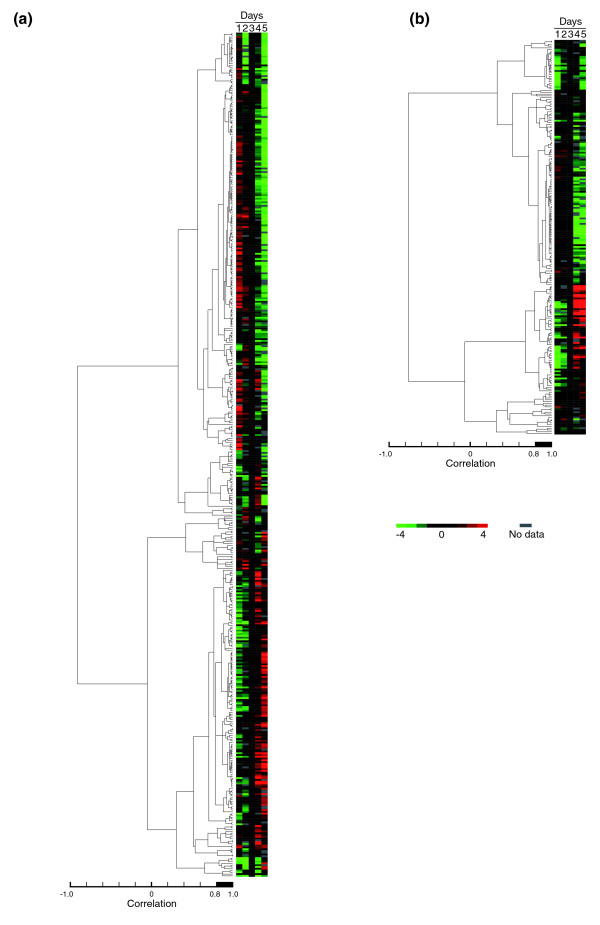
Average-linkage clustering of proteins quantified in the honey bee larvae. Proteins that were quantified in either or both the **(a) **tissue or **(b) **hemolymph for at least four out of five tested days were arranged by hierarchical clustering using software described in [46]. All expression values, shown relative to day 3 (= 0, black), have been natural log-transformed (>0, red; <0, green; no data, grey). These proteins, which have been manually annotated with a function and category, are calculated for enrichment within a node (results in Table 3) if the node correlation value is >0.8 (see thick bar on scale).

We used hierarchical clustering to further analyze the 522 proteins that were profiled in either or both the solid tissue (Figure 4a) and hemolymph (Figure 4b), followed by enrichment analysis according to manually assigned groupings. Clusters that satisfied the criteria (see Materials and methods) for significant enrichment are shown in Table 4 (complete dataset shown in supplementary Tables 7 and 8 in Additional data file 1 for tissue and hemolymph, respectively). Only a few functional classes of proteins were enriched in the same node, since expression profiles for some proteins exhibit biological variability that causes apparent inconsistency with the timecourse. Transcription and chromatin-associated proteins as well tRNA synthetases - clearly related by their tasks - shared node 376 (correlation 0.94) with pentose phosphate pathway and ubiquitylation enzymes. Energy storage and beta-oxidation proteins were both concentrated in node 434 (correlation 0.86, solid tissue). Protein turnover machinery, including ribosomes, protein folding, and proteasome, were all enriched in node 200 (correlation 0.84, hemolymph). Many of these clusters are also protein families already noted to show significant temporal regulation, such as energy storage proteins, ATP synthases, antioxidant proteins, and ubiquitylation enzymes. This indirectly suggests that suitable assignments were made during manual annotation and categorization, since their regulation patterns were grouped using independent methods.

**Table 4:** Enrichment analysis of protein classes following hierarchical clustering

Node number	Correlation	Proteins in this node	Protein class	Class total	Percent class enrichment
**Solid tissue**					
265	0.98	5	Helicase	3	67
277	0.98	8	Hormone synthesis	4	50
376	0.94	79	Transcription	3	100
376	0.94	79	Chromatin-associated protein	3	67
376	0.94	79	tRNA synthetase	3	67
376	0.94	79	Pentose phosphate pathway	4	50
376	0.94	79	Ubiquitylation	4	50
377	0.94	5	Food	6	50
411	0.90	108	Aldo-keto reductase superfamily	3	67
419	0.89	137	Proteasome	24	67
419	0.89	137	Antioxidant	16	50
419	0.89	137	Protein receptor	4	50
421	0.89	29	ATP synthase	10	60
427	0.88	34	Small molecule receptor	4	50
434	0.86	51	Energy storage	5	80
434	0.86	51	Beta-oxidation	8	50
438	0.83	7	Cuticle	7	57
439	0.83	146	Ras superfamily	10	50
					
**Hemolymph**					
128	0.97	35	Translation	7	57
150	0.96	6	Short-chain dehydrogenase family	4	50
180	0.93	21	Small molecule receptor	4	50
183	0.92	23	Food	8	63
183	0.92	23	Glycolipid metabolism	3	67
185	0.92	9	Ubiquitylation	4	50
190	0.89	63	Amino acid metabolism	8	50
197	0.86	29	Energy storage	4	100
200	0.84	81	Proteasome	10	90
200	0.84	81	Protein folding	20	60
200	0.84	81	Ribosome	31	81
203	0.82	21	Tricarboxylic acid cycle	4	75

Proteins that were manually categorized by function were subjected to average-linkage clustering (Figure 3). Separate analyses were done for solid tissue and hemolymph. Only proteins that were quantified for at least 4 out of 5 days and protein classes that had at least 5 members were considered for enrichment analysis. Classes with at least 50% enrichment in nodes with a correlation of >0.8 are considered significant and shown.

**Figure 5:**
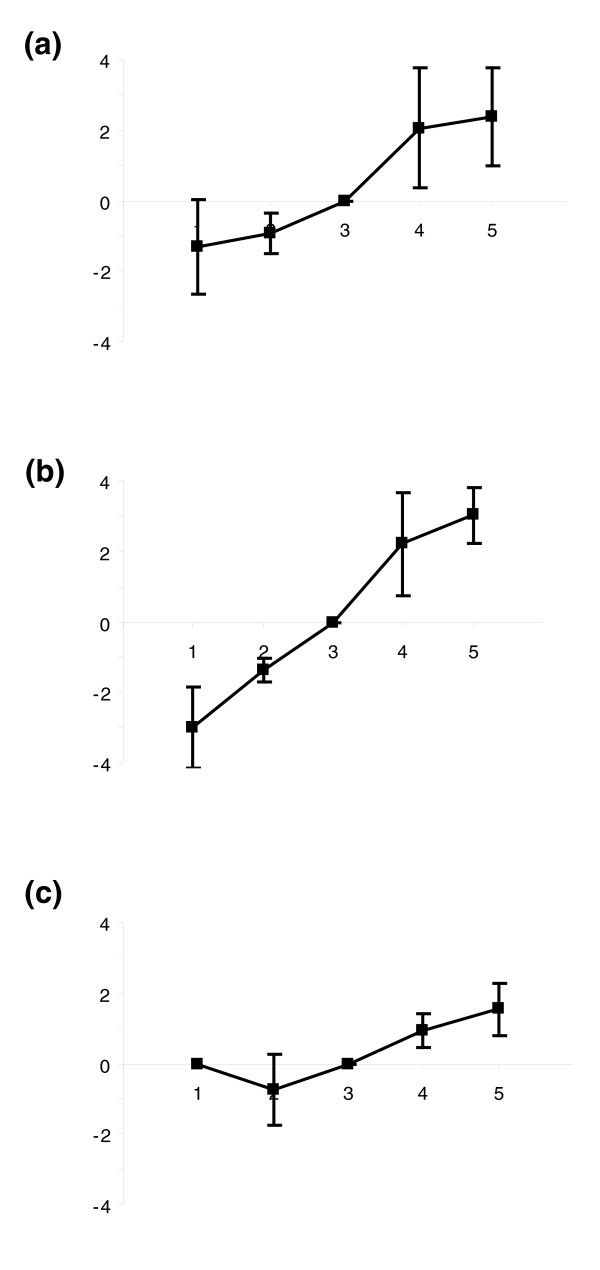
Expression profiles of four selected proteins during larval development. Expression levels (y-axis, expressed in natural log scale) over 5 days of larval growth (x-axis) are shown for 3 proteins discussed in the text: **(a) **imaginal disc growth factor [GenBank:66514614], **(b) **odorant binding protein 14 [GenBank:94158822], **(c) **apismin [GenBank:58585112]. Error bars represent one standard deviation.

Automated and semi-automated functional annotation and categorization effectively highlighted expression trends in large classes of proteins. With this approach, however, classes with only a few members or those where particular proteins have highly specialized function tended to fall below the significance threshold unless they were considered individually. In solid tissues, the levels of 86 proteins changed significantly (*p *< 0.05) over the tested period, accounting for 13% of all the quantifiable proteins in solid tissues. For example, levels of neuropeptide Y receptor increased 46-fold from day 3 to day 5. In the hemolymph, 66 of 481 (14%) quantified proteins changed significantly during the larval stage (*p *< 0.05). Most of these are intracellular proteins, yet the regulation of truly secreted proteins is frequently far more dramatic. An imaginal disc growth factor [GenBank:66514614] increased more than 13-fold from day 1 to day 5 (Figure 5a). Odorant binding protein 14 [GenBank:94158822] levels changed in a similar fashion, with the former displaying a 40-fold change over 5 days (Figure 5b). Antimicrobial peptide apismin [GenBank:58585112] (Figure 5c) and melanization enzyme prophenoloxidase [GenBank:58585196] expression were also positively correlated with age.

To our knowledge this is the first proteome-level description of honey bee larval development, so to gain additional insight, we compared our data with a previously reported developmental study of the fruit fly. While *Drosophila *and *Apis *are separated by 300 million years of evolution [2], *Drosophila *is nonetheless the closest highly studied phylogenetic neighbor of the bee. A whole body transcriptome study of the *Drosophila melanogaster *life cycle was published in 2002 [15], which included a list of genes that were significantly regulated during the larval period. After finding the protein homologs common to our study and the fruit fly larval dataset (34 in total), we calculated the slope of linear regression of expression trends for both organisms (the slope of the honey bee tissue and hemolymph profiles were averaged when needed; see Materials and methods). Slope values that have opposite signs or an absolute difference in slope of greater than 0.75 were considered dissimilar, amounting to 38% (13 of 34) of the proteins considered (Table 5; complete dataset with BLAST homolog search results in supplementary Table 9 in Additional data file 1). The most extreme slope reported for both organisms is for the hexamerin 70b protein (1.5 for bees and 1.6 for flies).

**Table 5:** Comparing expression levels in honey bee versus fruit fly larvae

Honey bee accession number	Protein description	Slope difference (honey bee minus fruit fly)	Expression trend: different or same?
GenBank:48126476	Translation: initiation factor 3f	0.01	Same
GenBank:110749015	Short-chain dehydrogenase family: oxidoreductase	0.22	Same
GenBank:110756656	Protein methylation: arginine methyltransferase	0.10	Same
GenBank:110759433	Ribonucleoprotein: ribonucleoprotein	0.10	Same
GenBank:110761364	Cytoskeleton: alpha-actinin	0.19	Same
GenBank:66547531	Pentose phosphate pathway: 6-phosphogluconate dehydrogenase	0.24	Same
GenBank:66509442	Peptidase: dipeptidyl aminopeptidase	0.02	Same
GenBank:110764347	Amino acid metabolism: enolase-phosphatase E1 (methionine salvage pathway)	0.50	Same
GenBank:110762382	Transcription: spermidine synthase	0.51	Same
GenBank:110763730	Antioxidant: glutathione S transferase	0.32	Same
GenBank:66504249	Uncategorized: protein kinase c substrate	0.33	Same
GenBank:110755309	Protein receptor: high density lipoprotein binding protein	0.33	Same
GenBank:110750855	Unknown function: unknown function	0.36	Same
GenBank:94158626	Electron transport chain: cytochrome p450	0.51	Same
GenBank:58585148	Energy storage: hexamerin 70b	0.15	Same
GenBank:48095159	Peptidase: serine protease	0.34	Same
GenBank:66524124	Peptidase: carboxypeptidase B	0.07	Same
GenBank:66509812	Peptidase: angiotensin converting enzyme	0.28	Same
GenBank:110762229	Peptidase: chymotrypsin	0.47	Same
GenBank:66510448	Glycolipid metabolism: beta-glucosidase (glucocerebrosidase)	0.10	Same
GenBank:110766932	Uncategorized: mannosidase, lysosomal	0.50	Same
GenBank:66513481	Ubiquitination: ubiquitin-activating enzyme E1	0.48	Different
GenBank:66522467	Uncategorized: juvenile hormone inducible protein	0.95	Different
GenBank:48104663	Protein receptor: protein kinase C receptor	0.60	Different
GenBank:110758189	Uncategorized: carboxylesterase	0.94	Different
GenBank:110756254	Ribonucleoprotein: ribonucleoprotein	0.67	Different
GenBank:66522232	Uncategorized: isochorismatase	0.43	Different
GenBank:66535270	Uncategorized: oxoacidtransferase	0.42	Different
GenBank:66521459	Membrane transporter: porin	0.59	Different
GenBank:110764660	Helicase: RNA helicase	0.56	Different
GenBank:110762902	ATP synthase: ATP synthase component	0.77	Different
GenBank:66525867	Small molecule carrier: solute carrier	0.82	Different
GenBank:58531215	Membrane transporter: translocase, ATP	0.87	Different
GenBank:110759569	Apoptosis: beta-hexosaminidase	1.14	Different

Genes in a life-cycle transcriptomic analysis of *D. melanogaster *[15] were compared to honey bee larval proteomics data in this report by finding homologs common to these studies. Significant matches (see Materials and methods for criteria) were assessed by comparing the slope values calculated between days 1 and 4: a protein is marked 'same' if the sign of the slope was the same and had a difference no greater than 0.75; otherwise, they are marked as 'different'.

## Discussion

The data presented here, at the level of the whole proteome, documents the dramatic changes occurring in developing honey bee larvae. The most striking, by far, is the 1,500-fold increase in weight over just 6 days [16]. In our proteomic analysis of the solid tissue, the most abundant organs are best represented, namely the fat body (accounts for 65% of the mass [17]), followed by the midgut and larval tubules. The hemolymph fraction reflects the secretory activities of all these tissues and also the molecules associated with intercellular communication and regulation. The presence of intracellular proteins suggest that hemolymph plays a major role in clearing apoptotic cells, in line with observations of the equivalent connective tissue in mammals (that is, blood) [18]. No dissection of specific larval organs was performed because many do not develop until the late stages, making direct comparisons of organ development by quantitative proteomics impossible.

We have found both automated (BLAST2GO) and semi-automated annotation (manual selection of descriptions provided by automated tools and manual categorization) to be very valuable for maximizing available information on an organism with otherwise very little functional annotation. While automated ontological methods were reliable and bias-free, outputs might be too generic (for example, 'ion binding' [GO:0043167]), or failing to accurately represent several very important protein families of the honey bee (for example, hexamerins and odorant binding proteins), highlighting the need for manual intervention. Cluster analysis is an indispensable tool for spotting expression trends, but given that the software for rigorous statistical enrichment analysis is designed specifically for popular model organisms such as mouse, worm, and yeast, the descriptive statistical approach used here was nevertheless able to provide credible insights about larval developmental biology or led to conclusions confirmed by other information.

The major behaviour during the larval stage is feeding as it prepares itself for the subsequent pupal stage when no feeding occurs. Based on various data acquired over the past century, it has been proposed that the larval fat body undergoes two phases, beginning with a high rate of protein synthesis and poor uptake of hemolymph substances, followed by a phase of low cellular synthesis and improved uptake and storage of hemolymph proteins [19]. Our data now allows us to clarify this model and provide molecular-level detail of these changes. One of the most remarkable events in a growing larva is the substantial synthesis of hexamerins and lipoproteins in the fat body, followed by their appearance in the hemolymph near the end of this developmental stage (reviewed extensively in [20]). While the age-dependent production of these abundant storage proteins is well known, here we provide evidence of a concomitant up-regulation of low copy transmembrane transporters [GO:0022857] that may facilitate the export, including a porin [GenBank:66521459]. Paradoxically, this astounding rate of protein production and export is paired with an opposite trend in protein synthesis machinery and accessories, which had been suspected in two reports in the 1960s [21,22]. Now we have evidence for these previous suggestions, including the clear age-associated decrease of more than 50 detected ribosomal subunits, coupled with an increase of two transcription repressors (although at *p *< 0.1 these did not satisfy significance criteria) to support this former notion.

Fat accumulation is an important purpose of the rapid larval growth, clearly indicated by the size of the fat body tissue relative to the whole organism, as well as the buildup of lipophorins. Lipids in larval food is only 4% by weight [23], meaning that *de novo *synthesis must account for the bulk of stored fat. Fatty acid synthase [GenBank:66515350] was one of the most abundant proteins throughout the entire tested period based on absolute protein expression estimates [24], yet to our surprise we did not observe significant temporal regulation in the expression of this enzyme with age. It is worth noting that 'fat body' is somewhat of a misnomer, given that it is involved in protein and glycogen storage, as well as fat [19,25]. To drive these endergonic biosynthetic processes, the demands for ATP must therefore be great. Not only do we observe significant age-associated increases in ATP synthase subunits, but also enzymes in energy-producing pathways such as the tricarboxylic acid cycle and the electron transport chain components. This may be attributed to an increase in mitochondria size or numbers; however, there are at least two reports that claim the number of mitochondria decreases as the larva approaches pupation in other insects [26,27].

Proteins with high copy number, including the many discussed above, are always the first to be investigated in any organism. The difficulties in studying proteins in honey bee larva have multiple sources: the abundant storage proteins broaden the dynamic concentration range, obscuring the rare proteins; the clean dissection of larval organs presents a technical challenge since the fat body is large and is difficult to remove; and finally, the lack of available antibodies against even the most common proteins makes many conventional biochemistry experiments, such as immunoprecipitation and western blotting, impossible. These reasons have especially hindered the study of fine larval organs such as the nervous system and low abundance proteins related to immunity or pathway regulation.

The ability of larvae to respond to external stimuli and internal regulatory cues increases with time, a trend that is clearly reflected in our data. For example, odorant-based communication has been observed in old larvae [28,29]. Odorant binding protein 14 [GenBank:94158822] was detected even on the first day after hatching, showing upregulation with age (Figure 5b). This suggests that younger larvae may have the capability to bind certain odorant molecules, but whether that translates into pheromonal communication is entirely speculative. The positive temporal regulation of antimicrobial peptide apismin [GenBank:58585112] (Figure 5c) and the melanization enzyme prophenoloxidase [GenBank:58585196] in the hemolymph, which have clear roles in defense [30-33], matches the observed susceptibility to diseases such as foulbroods of the young larvae, suggesting that one or both of these may be the factor responsible for successful defense against foulbroods in older larvae. However, a C-lectin [GenBank:110750008] and a complement factor [GenBank:66508940] actually have no observable expression trends, indicating that they may have alternative roles different from homology-based function predictions. The 46-fold increase of a neuropeptide Y receptor [GenBank:110764421], which controls appetite and fat storage, is reasonable given the feeding activity of the larvae. An imaginal disc growth factor [GenBank:66514614] increased by 40-fold over the course of the experiment (Figure 5a) presumably gears the larva for pupal development where specific limbs and organs will grow from imaginal discs containing highly differentiated cells.

Proteomics is generally a discovery method and is thus an excellent mechanism for hypothesis generation. We were able to find several peculiar proteins supported by a number of high quality mass spectra but no plausible explanation for its presence or degree of age-dependent regulation. A protein annotated as 'PREDICTED: similar to CG15040-PA' [GenBank:110749732] was consistently found only in the hemolymph of older larvae (up to 24-fold higher in 5-day old compared to 3-day old larvae), yet it has no likely homologs or discernable functional domains, bearing only a vague resemblance to a protein [GenBank:124512744] from *Plasmodium falciparum *3D7, found by PSI-BLAST [34,35].

## Conclusion

To study honey bees, individual, environmental, and social factors must be considered. The larval developmental stage has been shown to be a highly complex period of biochemical regulation. The proteomics data presented here are able to support a model for energy metabolism and storage, as well as reveal unexpected expression trends for proteins that respond to external and internal stimulus, such as pheromones, pathogens, and oxidants.

## Materials and methods

### Reagents

All salts and chemicals were of analytical grade or better and were obtained from Sigma-Aldrich (St. Louis, MO, USA) unless otherwise indicated. All solvents were of high performance liquid chromatography grade and were obtained from ThermoFisher Scientific (Waltham, MA, USA). The following materials were obtained as indicated: endopeptidase Lys-C from, Wako Chemicals (Osaka, Japan); porcine modified trypsin, Promega (Nepean, Ontario, Canada); loose ReproSil-Pur 120 C_18_-AQ 3 μm, Dr Maisch (Ammerbuch-Entringen, Germany); 96-well full skirt PCR plates, Axygen (Union City, CA, USA); fused silica capillary tubing, Polymicro (Phoenix, AZ, USA); 5 μl Microcap pipettes for hemolymph collection, Drummond (Broomall, PA, USA); soft forceps for holding bees, BioQuip (Rancho Dominguez, CA, USA); protease inhibitor mixture, Roche Applied Science (Basel, Switzerland); precast 4-12%, 1 mm thick NuPAGE Novex BisTris2 Gels, Invitrogen (Carlsbad, CA, USA).

### Obtaining larvae of known ages

Honey bee (*A. mellifera ligustica*) larvae were obtained from colonies kept at the University of British Columbia, Vancouver, BC, Canada. Samples were collected in the summer and early autumn. To acquire larvae of known ages, a queen was isolated on an empty frame of dark comb bracketed by two frames approximately 50% filled with honey and pollen for 16 h inside a nucleus colony with several hundred worker bees. The brood frame with newly laid eggs was then replaced into the original hive, along with the queen, workers and two supporting frames. The queen was separated from the newly laid eggs using a queen excluder to prevent additional eggs from being deposited. Three days after reintroducing the eggs into the colonies, larvae were collected for five consecutive days. In this system the maximum error in larval age would be 16 h. Empirical testing with shorter times did not yield enough eggs/larvae to sample the same population over all five days of development. Before proceeding with protein collection, all larvae were washed three times in phosphate buffered saline to reduce royal jelly contamination.

### Protein collection

For 1- to 3-day old larvae, hemolymph was collected by piercing the larval skin, taking care not to cause organ damage by avoiding deep cuts. For 4- and 5-day old larvae, hemolymph was collected by inserting a disposable 5 μl glass Microcap pipette two-thirds of the way down one side of the larva, drawing liquid by capillary action. All hemolymph samples were centrifuged for 10 minutes at 16,100 relative centrifugal force (r.c.f.) at 4°C to pellet cells and debris, which were added to the tissue samples. The tissues were homogenized by a bead mill using a tungsten bead in each 2 ml self-locking tube (Eppendorf, Hamburg, Germany) at 30 Hz for 5 minutes in 50 μl of phosphate buffered saline containing a protease inhibitor cocktail tablet solution (Roche) at 8 times the suggested concentration. Lysis buffer (100 μl of NP-40, and so on) was added before the sample was homogenized by 10 strokes through a syringe tipped with a 25 G needle. The sample was clarified for 10 minutes at 16,100 r.c.f. at 4°C and the pelleted debris was discarded. The Coomassie Plus Protein Assay (Pierce, Rockford, IL, USA) was used to determine protein concentrations of the tissue lysates and the clarified hemolymph according to the manufacturer's instructions before they were stored at -20°C until used.

### Denaturing protein gel electrophoresis

Tissue and hemolymph proteins were resolved on precast (Invitrogen) 4-12% NuPAGE gels (30 μg/lane) in reducing conditions with MES buffer according to the manufacturer's instructions. Blue-silver stain [36] was used to visualize protein bands.

### Sample preparation for mass spectrometry analysis

Larval tissue or hemolymph protein were aliquoted to provide 20 μg per sample before they were precipitated using the ethanol/acetate method as described [37]. The insoluble proteins were pelleted and temporarily stored at 4°C after a 10-minute centrifugation at 16,100 r.c.f. The ethanol supernatant was vacuum-dried, solubilized in sample buffer (3% acetonitrile, 1% trifluoroacetic acid, 0.5% acetic acid), and purified using the C_8 _flavor of STop And Go Extraction (STAGE) tips [38] to remove contaminants such as lipids, nucleic acids, and protease inhibitors. Bound proteins were eluted using 100% acetonitrile and vacuum-dried before adding 0.5 μl of 1.5 M Tris-HCl, pH 8.8. The bulk protein pellet and C_8 _purified proteins were digested using LysC and trypsin as described [37]. Peptides were desalted using C_18 _STAGE tips and the eluted solution was dried by vacuum centrifugation. For proteome profiling by relative quantification, binary analysis between timepoints was performed by chemical dimethylation of peptides from different timepoints using either light (CH_2_O) or heavy (CD_2_O) isotopologues of formaldehyde. For both the hemolymph and tissue samples, 3-day old larvae were used as a reference for all other timepoints, such that their peptides were always labeled with the opposing form of formaldehyde from days 1, 2, 4, and 5 before mixing the differentially labeled samples. Samples were fractionated on C_18_-SCX-C_18 _STAGE tips using a 10-step ammonium acetate elution gradient [39] and dried peptide samples were resuspended in 1% trifluoroacetic acid, 3% acetonitrile, 0.5% acetic acid prior to analysis on a linear trapping quadrupole-Orbitrap hybrid mass spectrometry (ThermoFisher Scientific, Waltham, MA, USA) as described in [3].

### Raw data processing

Following liquid chromatography-mass spectrometry analysis, peak lists were extracted from the raw data using Extract_MSN.exe (ThermoFisher Scientific) and DTA Supercharge [40] as described [41]. Results were searched using Mascot (v2.2) against a database containing the protein sequences of: Honey Bee Official Gene Set 1 [42], common exogenous contaminants (human and sheep keratins) and additives (porcine trypsin, lysyl endopeptidase C), the polyprotein of the deformed wing virus (common and often asymptomatic [43]), and the reversed sequences of all of the above as a decoy for reporting false discovery rates. The following Mascot parameters were used: trypsin (allowing up to one missed cleavage) or no enzyme specificity (in separate searches); carbamidomethyl as a fixed modification, variable modifications of dimethylation by both hydrogen isotopes at the peptides' amino termini and lysine ε-amino groups, 10 ppm peptide tolerance; 0.8 Da tandem mass spectrometry tolerance, and electrospray ionization-Trap fragmentation characteristics. Results were saved in Peptide Summary format with the 'Require Bold Red' option checked, applying a score cutoff corresponding to *p *< 0.05, which is 27 where results were limited to tryptic peptides, and 47 where no enzymes were specified. Since each sample was fractioned, generated files were combined using the in-house script Pickletrimmer.pl. MSQuant [40] was used to semi-automatically extract chromatographic peak volumes in both the light and heavy isotopologues of each detected peptide. Only peptides with an absolute calibrated mass error of <5 ppm were considered further. For protein quantification, the file was parsed (in-house script: QC_msqfa.pl) to obtain natural logarithm (Ln)-transformed heavy/light peptide volume ratios, which were median-normalized before they were averaged to calculate a relative protein ratio of day 3 larvae/day × larvae (where x = 1, 2, 4, or 5). From the three biological replicates of each binary comparison, proteins quantified with at least two quantified peptides from two or more replicates were averaged. Proteins whose relative quantities could be tracked for at least 4 of 5 days in either the tissue or hemolymph were considered to be profiled. For protein identification, the above peptides and unquantified sequences were extracted from MSQuant outputs. After removing redundant entries (in-house script: QC_remduplicate.pl), each was matched to their respective protein (in-house script: finalist.pl), excluding hits that were verified by equal to or less than two peptides of at least six or more residues. The false discovery rate was estimated by dividing the sequence-reversed proteins that failed to be eliminated after applying the above criteria.

### Automated protein annotation to Gene Ontology terms

All identified proteins were matched to GO [44] terms using BLAST2GO [12], following their standard procedure of performing BLAST searches for each protein (BLASTp, nr database, high scoring segment pair (HSP) cutoff length 33, report 20 hits, maximum e-value 1e-10), followed by mapping and annotation (e-value hit filter 1e-10, annotation cutoff 55, GO weight 5, HSP-hit coverage cutoff 20). After generating a directed acyclic graph (sequence filter 2, score alpha 0.6, node score filter 0) of molecular function terms (not shown), which groups specific terms into broader categories, ontologies on the third level of this graph were further analyzed by statistical testing (see below). The term 'protein binding' [GO:0005515] was omitted because this included the most number of proteins, most of which belonged under another more informative term.

### Semi-automated protein annotation and manual categorization

Protein descriptions were taken from several sources or tools, all of which are sequence homology-based derivations. Official protein names given in the Official Gene Set 1 [42] were used if the name was informative. BLAST2GO-derived descriptions were used where protein function was not clear from the official name (for example 'hypothetical protein'). If an appropriate name was still not derived, searches against the Conserved Domain Database (NCBI) were performed and considered matched for e-values <1e-10. As a final measure for matching a protein with a functional name, proteins descriptions were copied from Blast2seq [45] results (accessed via BLink in NCBI) if matches had >25% sequence identity and an e-value of <1e-10 over the aligned region. If none of these steps provided useful information, the protein was labeled and categorized with 'unknown function.' Proteins with descriptions but that did not fit under a specific category were classified as 'uncategorized' (supplementary Table 5 in Additional data file 1). Proteins that were not manually annotated are marked with 'NA' in the 'Description' column of supplementary Table 1 in Additional data file 1.

### Statistical analysis

To each class of manually assigned proteins, a pairwise, two-tailed *t*-test was performed using each protein in that class by taking the relative ratio in day 1 and comparing to day 5. Groups with *p *< 0.05 were considered to be temporally regulated, and their directionality of regulation was calculated by averaging the slopes of individual proteins within a group using day 1 and day 5 timepoints. Third-level GO molecular function terms were analyzed in the same manner, except all the proteins considered were quantified over all five days tested in at least one of the tissue or hemolymph datasets. To individual proteins, the same criteria for significance was used, taking values from each biological replicate as a data point in a pairwise comparison between the earliest and latest day the protein was quantified. We also performed average linkage clustering of the protein expression levels for proteins that were quantified over at least 4 days in either the tissue or hemolymph using Cluster and visualized by Treeview [46]. The grouping sizes ranged from 2 to 55 proteins. To normalize this variation, the number of proteins in a given class is reported as a percentage of the total class size (percent enrichment, using in-house script QC_nodeenrichment.pl). Only nodes that included at least 50% of all the proteins in that class and had a Pearson's correlation coefficient of greater than 0.8 were considered to be within the same cluster. Protein families with three or fewer members were included as part of the tree diagram, but were not considered for whether they formed a significant cluster.

### Comparison to Drosophila

Proteomic profiles resulting from this work were compared to the transcriptomic profiles of previously published *Drosophila *homologs [15] for the timepoints matching most closely to days 1 to 4 of the honey bee larval stage (h = 24, 49, 72, 96) for genes that were significantly regulated over this period (the fruit fly larval stage is shorter than that of bee by 1 to 2 days). BLASTp was used to find homologs in the Honey Bee Official Gene Set 1, which were defined as matches having e-values <1e-10 with at least 25% identity within the aligned region. Timepoints of the *Drosophila *dataset were normalized to the h = 72 timepoint and Ln transformed. To compare the expression trend between the two organisms, the slope of the line-of-best-fit for proteins (bees) or genes (flies) was calculated: expression trends with slopes that differed in signage or had an absolute difference of greater than 0.75 were considered to be dissimilar. Slopes whose absolute Pearson's correlation coefficient value was <0.5 were considered insignificant and, therefore, not considered. In instances where a significant slope could be calculated for a protein in both the tissue and hemolymph samples, the slopes were averaged.

## Abbreviations

GO: Gene Ontology; Ln: natural logarithm; r.c.f.: relative centrifugal force; STAGE: STop And Go Extraction.

## Authors' contributions

QWCT and LJF jointly conceived of the study, authored the scripts used in the data analysis and wrote the manuscript. QWCT conducted all the experimental work, mass spectrometric analysis and bioinformatics. LJF supervised the work and helped to troubleshoot throughout.

## Additional data files

The following additional data are available with the online version of this paper. Additional data file 1 includes supplementary Tables 1-10.

## Supplementary Material

Additional data file 1 Supplementary Table 1: relative quantification of bee larval proteome. Supplementary Table 2: Gene Ontology terms assigned to honey bee larval proteins. Supplementary Table 3: Gene Ontology categorization of proteins by molecular function using directed acyclic graphs. Supplementary Table 4: Gene Ontology 'molecular function' vocabularies assigned to proteins on level 3 of a directed acyclic graph. Supplementary Table 5: manually assigned protein function and functional class. Supplementary Table 6: average slope values of proteins within manually assigned functional classes. Supplementary Table 7: enrichment analysis of hierarchical clustering of proteins profiled from the honey bee larval solid tissue. Supplementary Table 8: enrichment analysis of hierarchical clustering of proteins profiled from the honey bee larval hemolymph. Supplementary Table 9: enrichment analysis of hierarchical clustering of proteins profiled from the honey bee larval hemolymph. Supplementary Table 10: peptide sequence data. Click here for file
